# Phenotypic techniques and applications in fruit trees: a review

**DOI:** 10.1186/s13007-020-00649-7

**Published:** 2020-08-06

**Authors:** Yirui Huang, Zhenhui Ren, Dongming Li, Xuan Liu

**Affiliations:** College of Mechanical and Electrical Engineering, Hebei Agricultural University, Baoding, 071001 China

**Keywords:** Phenotype, VIS–NIR spectroscopy, Spectral imaging, Thermal imaging, LiDAR

## Abstract

Phenotypic information is of great significance for irrigation management, disease prevention and yield improvement. Interest in the evaluation of phenotypes has grown with the goal of enhancing the quality of fruit trees. Traditional techniques for monitoring fruit tree phenotypes are destructive and time-consuming. The development of advanced technology is the key to rapid and non-destructive detection. This review describes several techniques applied to fruit tree phenotypic research in the field, including visible and near-infrared (VIS–NIR) spectroscopy, digital photography, multispectral and hyperspectral imaging, thermal imaging, and light detection and ranging (LiDAR). The applications of these technologies are summarized in terms of architecture parameters, pigment and nutrient contents, water stress, biochemical parameters of fruits and disease detection. These techniques have been shown to play important roles in fruit tree phenotypic research.

## Background

Plant phenotype describes the expression of plant traits. Phenotypes are studied at multiple levels, including cells, tissues, organs, individual plants and the whole orchard [1]. Plant phenotypic traits include but are not limited to plant height, biomass content, water state, and yield [2]. The expression of phenotypic traits is controlled by a large number of genetic factors. Therefore, accurate analysis of phenotypic traits is of great significance for the selection of dominant genes and marker-assisted selection [3, 4].

Fruit tree planting is an important part of agricultural production. In some cases, studies on fruit tree phenotypes have shown great reference value for accurate irrigation [5, 6], disease control [7, 8], and fruit quality evaluation [9]. In the past, digital callipers were used to measure tree height and crown diameter [10]. Physicochemical methods were applied to detect the pigment and nutrient content of blades, for example, the Kjeldahl method for the measurement of nitrogen (N) and oven drying method for the determination of moisture [11]. These methods are valuable but time-consuming and destructive to the plant.

With the development of technology, researchers began to develop rapid and non-destructive methods for the study of plant phenotypes. Spectroscopy has been found to be able to detect contents of biochemical substances [12]. Visible and near-infrared (VIS–NIR) spectrometers have become an effective instrument for spectral data collection because of their convenience [13, 14]. Some imaging devices are being used to speed up information acquisition [15, 16]. These techniques help to extend research from the level of single leaf to the level of the whole orchard, promoting the study of high-throughput phenotypes [3, 15, 17]. The present research not only focuses on the study of phenotypic information but also seeks to describe the spatial distribution of phenotypic traits. Light detection and ranging (LiDAR) scanning [18] can measure the spatial coordinates of monitoring points and provide reliable location information for describing the spatial variability in phenotypic traits. In addition, many efforts have been made to replace manual labour with automated mechanical equipment to build automated phenotypic platforms [2, 19].

**Fig. 1:**
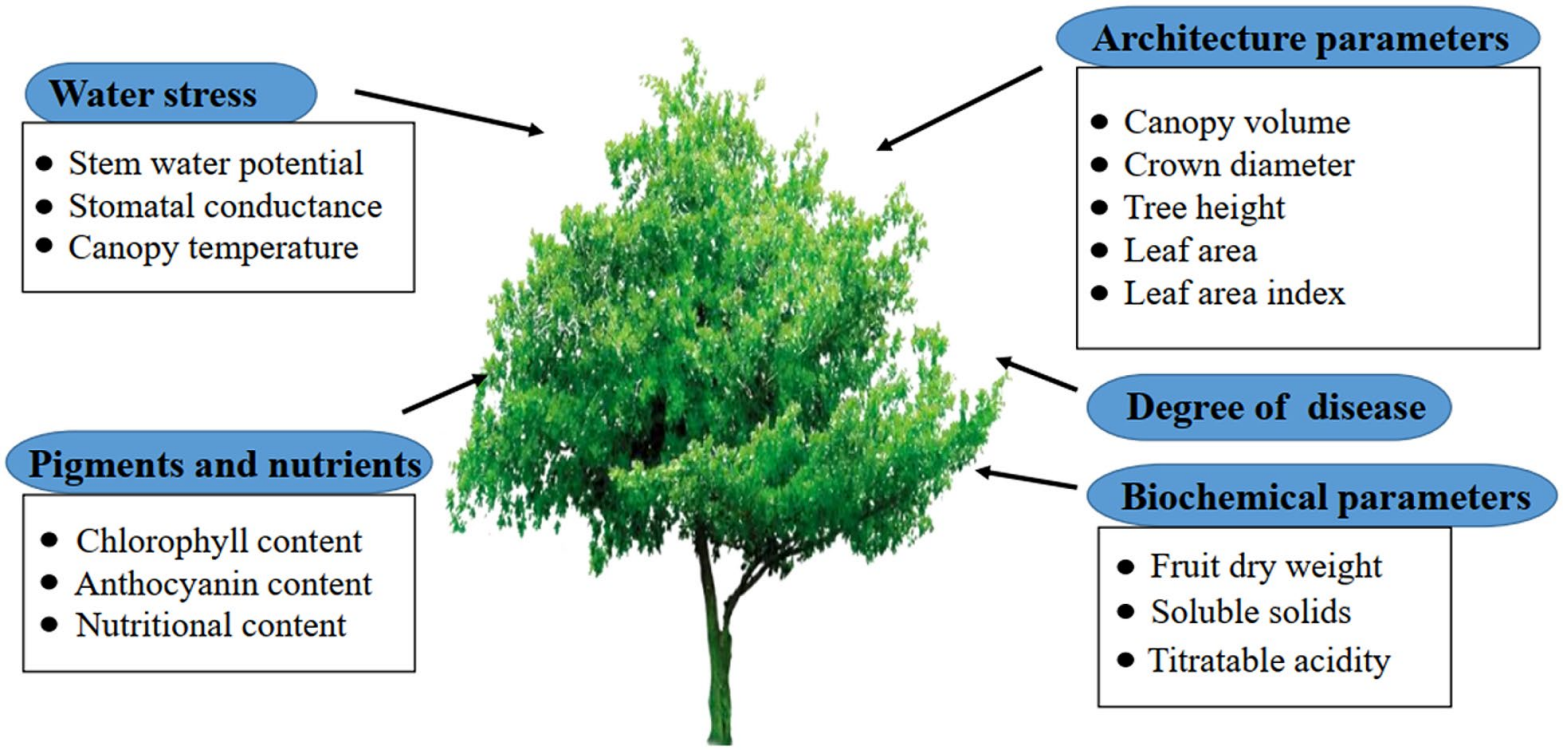
Five aspects and related phenotypic parameters of fruit trees

This paper aims to provide a comprehensive and in-depth review of the techniques for fruit tree phenotypic studies. We summarized the technologies and applications in the field around five aspects of fruit tree phenotypes (Fig. 1). The development trends and future challenges of phenotypic techniques are prospected at the end of this paper.

## VIS–NIR spectroscopy

VIS–NIR spectroscopy is a new non-destructive measurement technique. Based on the different reflection and radiation information of different substances in the same spectral band, VIS–NIR spectroscopy is widely used to detect chemical substances [20, 21], soil [22], minerals [23] and food [24]. The following articles in this section describe the principle of VIS–NIR spectroscopy and its application in the study of fruit tree phenotypes.

### The principle of VIS–NIR spectroscopy

Electromagnetic waves in a range of 400–2500 nm are often used in VIS–NIR spectroscopy [25]. Some of the groups in a substance, especially those containing hydrogen (C–H, O–H, N–H), absorb energy in VIS–NIR spectroscopy, resulting in changes in the reflected or transmitted spectrum [26]. When the substance content of the sample to be measured is different, various spectral curves will be generated. The spectral comprises broad bands arising from overlapping absorptions [27, 28]. Therefore, the corresponding relationship between spectra and the parameters to be measured can be established based on spectral features, to carry out quantitative analysis of parameters.

### The application of VIS–NIR spectroscopy

Portable spectrometer is a frequently used instrument in VIS–NIR spectroscopy studies, which can be applied for non-destructive detection. The sample can be measured directly with a light probe [27]. Moving the spectrometer to the sample location, rather than moving the sample to the laboratory, is the most convenient feature of portable spectrometers [28]. Different spectrometers have different spectral band ranges, and it is critical to select the suitable one for detection. Table 1 summarizes the application of VIS–NIR spectroscopy in fruit tree phenotypic studies in the field, and the details are described in the following sections.

**Table 1:** The applications of VIS–NIR spectroscopy in the study of fruit tree phenotypes

Applications	Species	Scale	Spectral range	Devices	Detected parameters	Evaluation parameters		Advantages	Limitations	References
						Indices	Performance evaluation			
Pigment and nutrient contents	Apple orchard	L	253–922 nm	Ocean Optics Fiber Spec	Chl		R = 0.91; RMSEP = 0.22	Easy to operate; non-destructive for leaves	More samples lead to longer testing time	[31]
		L	350–2500 nm	FieldSpec 3	Chl		R^2^ = 0.6213			[30]
	Vineyard	L	350–1000 nm	Ocean Optics USB2000	Chl		R^2^ = 0.8–0.9			[33]
					Carotenoid	SIPI	R^2^ = 0.49			
		L	350–2500 nm	FieldSpec3	Moisture		R^2^ = 0.96			[34]
					N		R^2^ = 0.95			
					Mg		R^2^ = 0.77			
Water stress	Citrus orchard	T	350–2500 nm	FieldSpec Pro	Water status		R^2^ = 0.89	Obtain the spectral value directly; not affected by sunlight conditions	Detection at the orchard level is limited	[36]
	Olive orchard	T	350–2500 nm	FieldSpec Pro	Ψ_leaf_	NDGI	R^2^ = 0.57; RMSE = 0.37			[37]
						MSI	R^2^ = 0.48; RMSE = 0.41			
		L			Ψ_leaf_	MSI	R^2^ = 0.45; RMSE = 0.72			
						NDWI	R^2^ = 0.45; RMSE = 0.75			
	Vineyard	L	350–2500 nm	FieldSpec4	EWT		R^2^ = 0.681			[39]
		T	325–1075 nm	FieldSpec Handheld2	Ψ_pd_	VARI	R^2^ = 0.79;			[38]
						NDGI	R^2^ = 0.79;			
		L	1600–2400 nm	Micro PHAZIR	Ψ_s_		R_cv_ = 0.77–0.93			[40]
					RWC		R_cv_ = 0.66–0.81			
		T	1100–2100 nm	NIR Spec	g_s_		R^2^ = 0.95			[41]
		T	1100–2100 nm	PSS 2120	Ψ_s_		R^2^ = 0.86			[42]
					g_s_		R^2^ = 0.66			
		T	1100–2100 nm	PSS 2120	Ψ_s_		R^2^ = 0.68–0.85			[43]
Biochemical parameters	Mango orchard	F	302–1148 nm	FieldSpec	TSS		R^2^ = 0.72	The method of data processing is simple	Interfered by the non-targets’ spectral information	[45]
					TA		R^2^ = 0.64			
	Vineyard	F	570–900 nm	PSS 1050	TSS		R^2^ = 0.95			[46]
					Anthocyanins		R^2^ = 0.79			
					Polyphenols		R^2^ = 0.43			

Note: In the “scale” column of the table, the fruit tree objects are divided into single leaves (L), individual trees (T) and fruits (F)

#### Detection of pigment and nutrient contents

Spectra are recorded with a sampling resolution of nanoscale by spectrometers so that hundreds or thousands of spectral variables will be obtained with each sample. Such a large amount of data often leads to the unreliability of the dependent variable prediction. Many variable selection methods have been developed to eliminate variables containing mostly noise, such as partial least squares (PLS), artificial neural networks (ANN), genetic algorithm (GA) and so on. For a detailed introduction to these methods, the reader is referred to [29].

Photosynthesis is an important process in the growth of green plants. Chlorophyll absorbs light energy and converts it into water and carbon dioxide via photosynthesis. Chlorophyll content in leaves can reflect the photosynthetic capacity and growth status of fruit trees [30]. An optical fiber spectrometer within the range of 500–1100 nm was used to determine the chlorophyll content in apple tree leaves [31]. Backward interval partial least squares (BiPLS) algorithm was applied to spectral data processing. From 1490 measuring bands, 71 bands with valid information were selected as input variables of the prediction model of chlorophyll content, and the value of R was 0.91. Wang et al. used the first derivative (FD) for the pre-process of spectral data [30]. Wavelengths of 530 nm, 581 nm, 697 nm and 734 nm were selected as sensitive wavelengths. The FDs were treated by the ratio and normalization methods, and four new parameters FD_530_, FD_734 _− FD_530_, (FD_734 _− FD_530_)/(FD_734_ + FD_530_), FD_697 _− FD_581_ were chosen to establish the PLS model. The PLS model exhibited an R^2^ value of 0.6213 for estimating the chlorophyll content in young apple leaves.

The vegetation index (VI) is the integration of spectral data from two or more bands after a certain mathematical transformation [32]. Researchers usually establish mathematical prediction models by calculating the spectral value of the sensitive wavelength or by calculating and optimizing vegetation indices defined in botany.

Zarco-Tejada et al. used an optical USB2000 spectrometer to detect the chlorophyll and carotenoid contents in grape leaves [33]. The spectrometer has a sampling interval of 0.5 nm which is beneficial for calculating narrow-band spectral indices. Several indices calculated within the range of 700–750 nm yielded good results with R^2^ value of 0.8–0.9 for chlorophyll estimation. The Structure Insensitive Pigment Index (SIPI) calculated by R_430_/R_680_ was used to estimate carotenoids with R^2^ = 0.49. The Photochemical Reflectance Index (PRI), calculated by (R_570 _− R_539_)/(R_570_ + R_539_), had a clear correlation with chlorophyll-carotenoid ratios. The results of the experiments above indicate that some spectral bands within the spectral ranges of green light (490–560 nm) and red light (620–780 nm) are significantly correlated with pigment contents.

For the estimation of nutrient contents, Ordonez et al. used FieldSpec 3 to characterize the components of vine leaves [34]. They applied functional nonparametric methods to establish prediction models. A curve was fitted to the discrete spectral data of different wavelength by the smoothing process. Moisture and nitrogen were predicted with high R^2^ values (R^2^ = 0.96 and R^2^ = 0.95, respectively). The relationship between the amount of Ca in leaves and reflectance is not sensitive, which may be due to the lack of comparative experiments on different fertilizers. The functional model use all the spectral features detected by the spectrometer instead of using characteristic wavelength values so that the utilization rate of hyperspectral information is improved [35].

The accuracy of portable spectrometers is lower than that of laboratory spectrometers, but portable spectrometers are affordable, small in size, and easy to use, which are useful for non-scientists [28]. VIS–NIR spectroscopy could be an effective diagnostic tool for predicting nutrient deficiencies in fruit leaves [34], and implementing reasonable fertilization management.

#### Detection of water stress

The evaluation of water stress aims to determine the status of the water deficit in orchards. Before water stress has a significant impact on fruit trees, reasonable irrigation will reduce the degree of damage to trees. Stomatal conductance (g_s_) and stem water potential (Ψ_s_) are two representative indicators reflecting vegetation water status.

FieldSpec Pro with a spectral range of 350–2500 nm was applied to detect water status in citrus trees [36]. The study found significant differences in the monthly mean reflectance of the citrus canopy in summer (~ 22%) and winter (~ 15%), which indicated that canopy reflectance can be used to provide the water condition of citrus trees. Rallo et al. used a portable spectrometer to collect the spectral information of the paraxial position of the blade on a one-year-old shoot in olive groves [37]. The spectrometer was placed on an aluminium mast mounted on a horizontal arm at a distance of 1 m above the canopy. The angle of view of the sensor was vertically downward, which covered an area of approximately 0.12 m^2^ of the canopy. The results showed that optimized indices, the Normalized Difference Greenness Vegetation Index (NDGI) and the Normalized Difference Water Index (NDWI), had strong correlations with leaf water potentials (Ψ_leaf_).

For field spectrometers, the spectral resolution can be 2 nm, which means that there are many closely spaced bands in the same property. The selection of the most suitable wavelength for mathematical modelling will improve the accuracy of the phenotypic parameter estimation. Rallo et al. utilized NDWI and Moisture Spectral Index (MSI) to evaluate the water content [37]. In the calculation, they found that the central wavelength of the NIR band should be selected at 715 nm for the estimation at the canopy level and 750 nm for the estimation at the leaf level, which are lower than the standard of 858 nm. Pôças et al. also analysed the optimal wavelength when evaluating the water status in a vineyard [38]. The wavelengths 520 nm (blue), 539 nm (green) and 586 nm (red) were selected as the best wavelengths for the calculation of the Visible Atmospherically Resistant Index (VARI). González-Fernández et al. used FieldSpec 4 to detect the water status in a vineyard [39]. Spectral acquisition experiments were carried out at both the leaf and canopy levels. The canopy measurements were made at nadir and at 0.30 m above the canopy. Researchers used continuous removal analysis to highlight the absorption and reflection characteristics of the spectral curves. In relation to the equivalent water thickness of the blade, the band area at 1450 nm contributes to a higher correlation than that at 1200 nm or 1950 nm.

The advent of field spectrometer allowed spectral detection in the field, but the instrument is too large and heavy for workers. The application of handheld spectrometers makes it possible to measure without complex optical fiber connections and backpacks. Diago et al. used a handheld digital transform spectrometer working in the spectral range of 1600–2400 nm to detect water stress at the leaf level in different vineyards [40]. Reliable predictions of Ψ_s_ and leaf relative water content (RWC) were achieved from regression models. In addition, handheld spectrometers can also be used at the canopy level. Similar to the way portable spectrometers are used in canopy level experiments, the spectrometer sensor would be maintained above the canopy, and the angle of view was vertically downward. The detectable canopy diameter should be smaller than the canopy diameter to eliminate interference from soil [38].

Using portable and handheld spectrometers in the field avoids the destruction of vegetation and improves the collection speed; however, there are still challenges in terms of time and manpower for collecting large amounts of data. Realizing the automation of data collection is the key to researches on high-throughput fruit tree phenotypes. Diago et al. installed a VIS–NIR spectrometer on an all-terrain vehicle, and the sensor head was mounted at a height of 1.40 m above the ground. The spectrometer was fixed at a distance of 25–50 cm from the canopy [41]. When detecting the spectral information of grape leaves, the vehicle needed to be stopped first. In the following year, the same team, using a similar device, performed spectral measurements while the vehicle was in continuous motion [42]. In this case, the original spectral information obtained will contain information about voids, wood, metal, etc. To filter information about the canopy from the original data, static blade characteristics should be collected before the experiment. The spectral detection instrument, mounted on a vehicle to operate contactless detection, was called on-the-go spectroscopy [41]. Instead of human workers, the vehicle carries the spectrometer for movement. The automation of acquisition tools greatly improves the efficiency of data acquisition.

Compared with traditional methods, the application of VIS–NIR spectroscopy to evaluate phenotypic information can reduce the damage to fruit trees. For the leaf level, the detection is rapid and effective. For the canopy level, a spectrometer on a tripod is used to detect spectral information for individual trees. The emergence of on-the-go spectroscopy speeds up data collection and contributes to the study of high-throughput phenotypes. On-the-go spectroscopy has the ability to take measurements in multi-rows and enables mapping of the variability in the fruit tree water status in orchards, which is of great value for formulating reasonable irrigation measures. The orchard could be divided into differentiated zones according to the variability in the water status. Different watering schedules and doses for different zones can greatly reduce water waste [43], which is responding to the policy of sustainable development.

#### Detection of biochemical parameters of fruits

Chlorophyll, carotenoids, total soluble solids (TSS) content and titratable acidity (TA) are biochemical parameters of fruit, and they will change gradually with the fruit growth [44]. Accurate prediction of biochemical parameters will contribute to judging the maturity of fruit and determining whether it is suitable for harvest. This section mainly focuses on the detection of the fruit in living conditions.

Elsayed et al. used a handheld spectrometer with wavelengths of 302–1148 nm to test the biochemical parameters of mangoes [45]. The optical fiber probe was placed at a zenith angle of 30 degrees and 0.15 m above the mango fruit for non-contact detection. A contour map was made for the coefficients of determination of all biochemical parameters of mango fruits with all possible wavelength (302–1048 nm) combinations. Twelve wavelengths (810, 780, 760, 750, 730, 720, 710, 686, 620, 570, 550 and 540 nm) were selected to estimate TSS (R^2^ = 0.72) and TA (R^2^ = 0.64). The results of partial least square regression (PLSR) models revealed that the newly developed index (NDVI − VARI)/(NDVI + VARI) (NDVI: Normalized Difference Vegetation Index) showed a close association with chlorophyll meter readings (R^2^ = 0.78).

In addition to the detection of specific points, on-the-go spectroscopy has successfully realized continuous spatial detection. A PSS 1050 spectrometer operating in the 570–990 nm spectral range was installed on an all-terrain vehicle [46]. To align the detection probe to the position of the grape cluster, the height of the spectrometer sensor was adjusted to 0.8 m above the ground, the angle was adjusted to level, and the sensor had a distance of 0.3 m from the canopy. According to the spectral characteristics of the grape clusters obtained artificially, the threshold was constantly adjusted to separate out the true berry spectrum from the raw data. TSS was estimated with R^2^ value of 0.95.

On-the-go spectroscopy is proven to be feasible for the detection of canopy water stress and fruit biochemical parameters in vineyards. It should be noted that the canopy of vineyard is continuous and different from that of citrus or apple orchards. Extracting effective spectral information is the key to data processing in the application of on-the-go devices in orchards with discontinuous canopies. The calculation of spectral indices based on sensitive wavelength is convenient, but the spectral characteristics of other wavelengths are neglected in this process. Establishing prediction models for fruit tree phenotypes based on the full spectral information will greatly improve the utilization of spectral information, to obtain results with high accuracy.

## Digital photography

With the rapid development of digital computer and image processing technology, digital photography is becoming increasingly popular in scientific research and daily life. The approach of obtaining plant colour and spatial information from digital images has been successfully applied in the study of plant phenotypes [47–49].

### The principle of digital photography

The charge-coupled device (CCD) is a semiconductor device, which is applied in imaging technology as an image capture component. CCD can directly convert optical signals into analogue current signals and realize image acquisition and reproduction through analogue-to-digital conversion. With the continuous progress of chip technology, complementary metal oxide semiconductor (CMOS) has gradually replaced CCD with the advantages of low energy consumption and moderate price [50]. Digital photography is an image acquisition technology for colour communication [51]. Digital images can be taken instantly and easily transmitted and edited. Depending on these advantages, digital photography is rapidly applied in scientific research. This section mainly focuses on the application of digital photography in the study of fruit tree phenotypes in the field.

### The application of digital photography

In the study of fruit tree phenotypes, digital photography is mainly used for the determination of canopy structural and biochemical parameters. Fisheye photography and digital cover photography are two techniques with different lenses, both cameras are useful in plant phenotypic analysis, especially in the determination of leaf area index (LAI) [52, 53]. A summary of applications of digital photography in fruit tree phenotypic studies is given in Table 2.

**Table 2:** The applications of digital photography in the study of fruit tree phenotypes

Applications	Species	Scale	Devices	Detected parameters	Evaluation parameters		Advantages	Limitations	References
					Indices	Performance evaluation			
Architecture parameters	Apple orchard	T	Nikon FC-E8 fisheye lens	LAI; PAI			Low cost; high accuracy; especially suitable for the determination of LAI	Susceptible to uneven light and overlapping blades	[55]
		T	CID CI-110 fisheye lens	LAI		Error = 13%			[56]
		T	Digimax A503 Samsung	LAI		R^2^ = 0.85; RMSE = 0.22			[61]
	Almond orchard	T	Nikon FC-E8 fisheye lens	LAI	NDVI	R^2^ = 0.88			[57]
					NDWI	R^2^ = 0.91			
	Vineyard	T	Digimax A503 Samsung	LAI		R^2^ = 0.97; RMSE = 11.5%			[62]
		T	Canon EOS 60D	LA		R^2^ = 0.93; RMSE = 3.0%			[63]
	Citrus orchard	T	Canon EOS 6D	C_v_					[64]
Biochemical parameters	Mango	F	Kodak D5100	Chl-a	(NDVI − VARI)/(NDVI + VARI)	R^2^ = 0.71	RGB images can reflect colour information well	The evaluation accuracy is not high enough	[45]
				Chl-t		R^2^ = 0.71			
				Chl-b	(R − B)/(R + B)	R^2^ = 0.57			
				Carotenoids		R^2^ = 0.53			
				TSS		R^2^ = 0.57			
				TA		R^2^ = 0.59			

Note: In the “scale” column of the table, the fruit tree objects are divided into individual trees (T) and fruits (F)

#### Detection of architecture parameters

Digital image has high image resolution, which is valuable for the calculation of canopy architecture parameters. The architecture parameters include tree height, crown diameter, crown volume (C_v_), leaf area (LA) and LAI. LAI is the total one-sided area of leaf tissue per unit ground surface area [54], it can be regarded as a reliable basis for pruning branches and leaves, to improve light transmittance and promote the growth of branches and leaves.

Digital hemispherical photography (DHP) is a type of digital imaging with fisheye lenses. Pictures are usually acquired from beneath the canopy towards the zenith, or from above the canopy looking downward in phenotypic research. Jonckheere et al. reviewed the methods for indirect measurement of LAI by using DHP technology [47]. The advantage of using DHP is that several available commercial integrated instruments have been invented for LAI estimation for the image processing to reduce the intervention of operators. Each system contains a specific imaging device and a free analysis software [54, 55].

Illumination condition and shooting distance are intuitive factors affecting image quality. To improve the accuracy of LAI estimation, Knerl et al. conducted multiple experiments to determine the optimal shooting environment [56]. Two kinds of coloured anti-hail nets (blue and pearl nets) were artificially created over the apple trees to mimic uniform overcast and ideal clear sky conditions. The images were taken at distances of 10, 20 and 40 cm respectively, above the ground under the canopy. The OTSU algorithm was selected for threshold prediction. The processing result showed that when the images were taken for a tree group from approximately 10 cm away from the ground in a net-free environment, the predicted LAI had the smallest deviation from the destructive LAI. In the threshold selection, Zarate-Valdez et al. [57] discovered that the contrast threshold for distinguishing leaves from the sky needed to be verified many times to generate reliable LAI.

Digital cover photography (DCP) has become a substitute for DHP with the advantage of high resolution. DCP uses a narrow field-of-view lens aimed at the zenith for imaging [58]. Compared with hemispherical photography, DCP is not sensitive to image exposure; however, there is a lack of software for digital cover image processing automatically [53, 59].

To improve the automation of the analysis methods for cover images, Fuentes et al. used a script written in MATLAB 7.4 to replace the manual technique for LAI estimation of eucalyptus woodland [60]. The developed script can directly connect the laptop to the digital camera to obtain cover photographs and LAI analysis in real time. In subsequent research, the script was also applied to determine the LAI of fruit trees in apple orchards and vineyards [61]. In addition, Fuentes et al. added an automated module to the original code, and frames (images) were extracted from videos by commands from the Image Analysis Toolbox [62]. The new script could be successfully applied to analyse the LAI of grape trees from videos.

The development of specific software and automation programs for hemispherical and digital cover images provides an accurate and rapid method for the determination of the LAI of fruit trees. However, some studies have indicated that an ordinary consumer digital camera without special sensors can also be used to detect phenotypic information of fruit trees with its ability to perceive colour information.

Taking advantage of high resolution of digital images, Klodt et al. presented an image segmentation method based on colour information [63]. Image pairs with overlapping information were obtained from different locations for each plant. The depth map was constructed by calculating the depth information according to the displacement of the target point in image pairs. Fruits, leaves, stems and background in the image were segmented according to the colour information. According to the depth information, the pixel size in the segmented image was weighted to calculate the vine leaf area. This method has been successfully applied to the calculation of LA and fruit-to-leaf ratios in vineyards.

In addition, the structure from motion (SfM) of orchards can be carried out by using digital images, which is convenient to detect the canopy volume of fruit trees. Haris et al. obtained low-altitude images of an citrus orchard by UAV and generated a 3D map of the orchard [64]. They proposed a method to divide the 3D image of trees into a collection of voxels for the estimation of canopy volume. A voxel was a 3D array that represented the depth of an image. The canopy volume was calculated by calculating the number of voxels occupied by each canopy and the volume of each voxel. Canopy volumes of 78 trees can be measured in 15 min by this method. The efficiency has been significantly improved compared to the manual measurement (10 min for each tree measured).

LAI is a dimensionless quantity representing the canopy and a significant parameter for quantitative analysis of ecosystem productivity [54]. In traditional measurement approaches, the LAI is equivalent to the cumulative leaf area of the leaf fall period in a known collection area [65]. Although this calculation method obtains the most realistic results, it needs to go through a long process. Studies have shown that digital photography is a reliable method for the measurement of LAI. Moreover, the estimation of LA and C_v_ by digital imaging can help farmers monitor the growth condition of fruit trees.

#### Detection of biochemical parameters of fruits

The colour digital image represented by red, green and blue components is called RGB image [50]. RGB images can accurately reflect the colour information of the target. Extracting the three colour components of R, G and B is the key to RGB image processing [66]. Some vegetation indices (VIs) expressed by colour components can be used to predict biochemical parameters of fruits.

Elsayed et al. proposed a method for the determination of the chlorophyll content of mango fruits by the VARI and the NDVI calculated by (R − B)/(R + B) and (G − R)/(G + R − B), respectively [45]. According to the PLSR models, the newly developed index (NDVI − VARI)/(NDVI + VARI) showed close and highly significant associations with chlorophyll a and chlorophyll t (the sum of chlorophyll a and chlorophyll b). In addition, the index (R − B)/(R + B) was a good predictor of TA.

The determination of phenotypic information of fruit trees by digital photography results in no damage to fruit trees, and its ability to view images instantly without rinsing film brings great convenience to data acquisition. In addition, the segmentation of fruit trees and backgrounds based on colour information provides a new method for image processing.

## Multispectral and hyperspectral imaging

Spectral imaging is a technique used to divide the breakdown of ground object electromagnetic radiation into several narrow spectral segments and obtain information for different bands of the same target at the same time by means of photography or scanning. Spectral imaging sensors can detect information in spectral bands beyond the visible range, such as infrared wavelengths, providing researchers with additional raw data [67].

### The principle of multispectral and hyperspectral imaging

The visible to long-wave infrared spectral spectrum (0.4–14 µm) is commonly used in scientific research. The electromagnetic waves in this band can be divided into four categories: VIS band (400–700 nm), NIR band (700–1000 nm), short-wave infrared band (1000–2500 nm) and long-wave infrared band (7.5–14 µm) [68].

Spectral imaging is a technology can simultaneously obtain the two-dimensional spatial information and one-dimensional spectral information of the target, covering a variety of disciplines such as spectroscopy, optics, computer technology, electronics technology, and precision machinery [69]. Multispectral imaging adopts parallel sensor arrays and detects a small amount of reflection over broad wavelength, which is generally composed of three to six discontinuous bands. Hyperspectral imaging detects reflection of hundreds of continuous spectral bands, and the band widths are narrower than the widths of multispectral bands [5]. Therefore, hyperspectral imaging can yield in-depth information about specimens that are easily lost in multispectral imaging.

### The application of multispectral and hyperspectral imaging

**Fig. 2:**
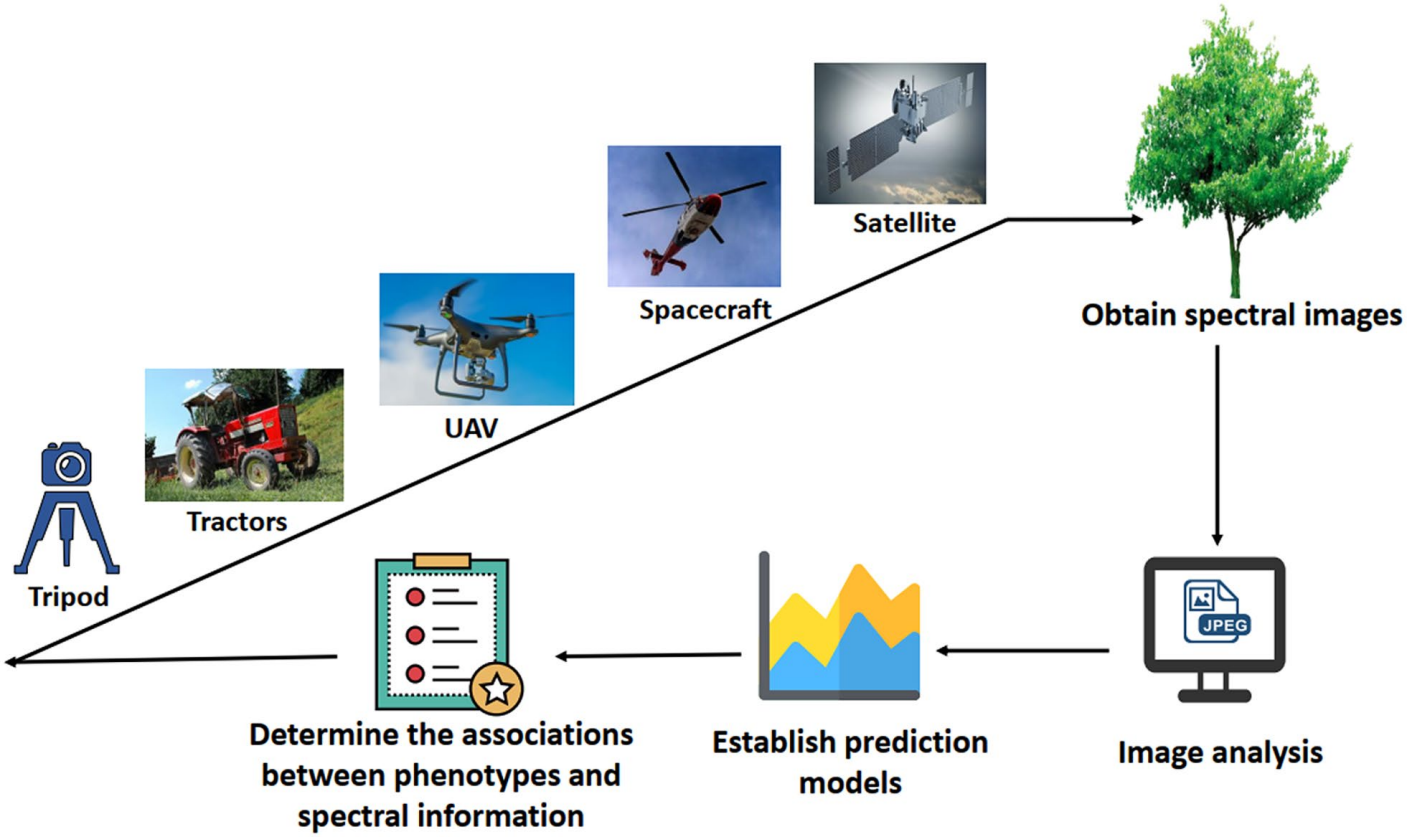
The acquisition and processing methods of multispectral and hyperspectral imaging in the study of fruit tree phenotypes. The analysis has four steps, as shown in the figure

As computer technology and new optical equipment have evolved, many kinds of multispectral and hyperspectral imager devices have been developed. The spectral imager needs to be stable during image acquisition. Darkroom and halogen lamps are usually designed for spectral image acquisition in the laboratory [16, 70]. Ground-based spectral imaging system is suitable for experiments in the field. The tripods and vehicles are used as the bearing device for the camera [36, 71]. To quickly obtain spectral data of the whole orchard, an unmanned aerial vehicle (UAV) was applied for imaging [72]. As the UAV flies along the route path, the spectral camera takes continuous images at regular intervals [19]. In addition, spectral cameras mounted on manned spacecrafts and satellites can capture spectral images on a large scale. The acquisition and processing methods of multispectral and hyperspectral imaging in the study of fruit tree phenotypes are shown in Fig. 2. The application of spectroscopy in phenotypic studies has a long history [16], and this review mainly focused on the research over the last 5 years. A summary is listed in Table 3, and some details are described in the following section.

**Table 3:** The applications of multispectral and hyperspectral imaging in the study of fruit tree phenotypes

Applications	Species	Scale	Spectral range	Devices	Detected parameters	Evaluation parameters		Advantages	Limitations	References
						Indices	Performance evaluation			
Architecture parameters	Olive orchard	O	B, G, R, red edge, NIR	Tetracam mini-MCA-6	Canopy area		R^2^ = 0.94; RMSE = 1.44 m^2^	Suitable for the information acquisition of the whole orchard canopy	High cost; difficult to accurately detect blade orientation	[77]
					Tree height		R^2^ = 0.90; RMSE = 0.24 m			
					C_v_		R = 0.65			
		O	IR	Panasonic Lumix DMC-GF1	Tree height		R^2^ = 0.22			[75]
					Crown diameter		R^2^ = 0.58			
	Vineyard	O	G, R, NIR	ADC-Snap	Tree height	NDVI				[73]
Pigment and nutrient contents	Apple orchard	O	VIS, red edge, NIR	Multispectral imager	Chl	NDVI	R^2^ = 0.667; RMSE = 0.178	Wide spectral band range; real-time monitoring of a large area	Not suitable for parameter determination of single blades	[32]
	Citrus orchard	O	490–950 nm	Mini-MCA12	Total N		R = 0.6469; RMSEP = 0.1296			[82]
					Total soluble sugar		R = 0.6398; RMSEP = 8.8891			
					Starch		R = 0.6822; RMSEP = 14.9303			
		O	400–885 nm	Micro-hyperspec VNIR model	Chlorophyll fluorescence	FLDn	R^2^ = 0.72			[80]
	Pear orchard	O	550–810 nm	Tetracam Micro-MCA	Leaf%N	M3CI	R^2^ = 0.67; RMSE = 0.24			[83]
	Vineyard	O	515, 530, 570, 670, 700, 800 nm	Multispectral sensor	Carotenoid content	R_515_/R_570_	R^2^ = 0.43			[78]
			400–885 nm	Micro-hyperspec VNIR model		R_515_/R_570_	R^2^ = 0.48			
						R_515_/R_570_, TCARI/OSAVI	R^2^ = 0.42; RMSE = 0.87			
Biochemical parameters	Mango orchard	T	390.9–887.4 nm	Resonon Pika II	DM		R^2^ = 0.64	Quick detection; time saving; No need for chemical treatment	Advanced image processing techniques are required	[71]
		T	390.9–887.4 nm	Resonon Pika II	Yield		R^2^ = 0.83			[88]
	Vineyard	T	400–1000 nm	Resonon Pika L	TSS		R^2^ = 0.91			[87]
					Anthocyanin concentration		R^2^ = 0.72			
Diseases detection	Avocado orchard	O	390–520 nm; 470–570 nm; 670–750 nm	Modified Canon	Distinguish laurel wilt disease	B/G		Suitable for disease detection over large scales; not influenced by the variation in agronomic characteristics	Lack of ability to diagnose disease	[94]
		O	580, 650, 740, 750, 760, 850 nm	Tetracam mini-MCA-6	Distinguish laurel wilt disease	TCARI_760–650_				[95]
						NIR/G				
		O	560, 660, 830 nm	ADC Micro	Identify white root rot disease	NDVI	Accuracy is 82%			[96]
	Olive orchard	O	400–885 nm	Micro-Hyperspec VNIR	VW severity levels	FLD3	Accuracy is 79.2%			[92]
	Almond orchard	O	400–885 nm	Micro-Hyperspec VNIR	Red leaf blotch development	FLD2				[93]
						Chl_a+b_				
						Carotenoid				

Note: In the “scale” column of the table, the fruit tree objects are divided into individual trees (T) and the whole orchard (O)

#### Detection of architecture parameters

It is a useful method to establish digital terrain models (DTMs) of orchards by using low-altitude images and global positioning system (GPS) for the identification of canopy architectural features. DTM is an ordered numerical array that describes the spatial distribution of various information on the Earth’s surface. DTM without ground objects is referred to as digital elevation model (DEM), and DTM with ground objects is known as digital surface model (DSM). Agisoft PhotoScan is a special computer vision software that can automatically identify and match features of multiple images and build a DTM of the research area by combining ground control point parameters, GPS positioning and internal parameters of the camera. Matese et al. measured the canopy height of vine rows by constructing DSMs and DTMs [73]. Images of the vineyard in the R, G and NIR bands were obtained by a multispectral camera. The canopy height model, representing the relief of the vine row surface, was obtained by subtracting DTM from DSM. The estimated canopy height is approximately 0.5 m lower than the actual canopy height. They also built a NDVI map of the vineyard and found a good correlation between NDVI values and canopy heights in the aera with high canopy height. This finding provided an idea for estimating canopy architecture parameters using VIs.

Pixel-based segmentation results are prone to produce salt and pepper noise because that the size of a single pixel is much smaller than the detected object. Therefore, object-based image segmentation techniques are increasingly used in phenotypic studies [74]. Díaz-Varela used multi-resolution segmentation and supervised classification algorithms to segment the olive canopy and background from UAV images captured by a modified RGB camera [75]. The segmentation of single crowns is performed by the watershed algorithm. The canopy was isolated by a segmented contour line, and tree height was retrieved from DSM based on the identification of local maxima. As a result, crown diameter was predicted with R^2^ = 0.58 and R^2^ = 0.22 in discontinuous and continuous canopies, respectively, and tree height was estimated with R^2^ = 0.07 and R^2^ = 0.58. Koc-San et al. proposed circular Hough transform algorithm to extract citrus trees from DSM [76]. Combined with the specific canopy size and spacing, the images were processed by threshold analysis, median filtering and edge detection to obtain the edge of the tree shadow. Then, according to the azimuth of the sun, the circular shadow was moved to obtain the exact boundary of tree crowns. This method is of great value for distinguishing tree crowns from other plants which have similar radiation conditions. A conclusion can be drawn from this result that circular Hough transform algorithm is available for the identification and feature extraction of fruit trees with green, round and compact features. Torres-Sánchez et al. classified vegetation and bare land area based on vegetation index values. The DSM layer was applied to separate trees with the surrounding soil according to the difference in height [77]. This method provides a good estimation of tree height (R^2^ = 0.90) and canopy area (R^2^ = 0.94). Considering the spatial characteristics and contextual features, the object-oriented classification method takes spatial pixel cluster as the classification feature instead of a single pixel, that is suitable for high resolution image processing.

RGB images have high spatial resolution, which is conducive to the accurate acquisition and matching of ground control points in the modelling of DTMs. The spatial resolution of multispectral images is slightly lower than that of RGB images, so it is easy to lose similarities in the matching process of multispectral images. However, multispectral cameras can detect reflection beyond RGB bands, which is valuable in image segmentation for vegetation and background pixels with significant contrast in infrared bands. The segmentation of canopy and background pixels is an important part of image processing, an algorithm that is suitable for the distribution characteristics of fruit trees will help to obtain ideal results. In summary, it is necessary to find a balance between the accuracy of DTMs and the complexity of image processing to select the appropriate technology for phenotypic research.

Biomass is one of the most important parameters of canopy management. Architecture parameters can be used as the basis for assessing biomass [73]. The estimation of architecture parameters of fruit trees with UAV imaging at orchard level, allows creating maps of orchard heterogeneity and observing zones with different tree sizes, which provide a prerequisite for precision agriculture.

#### Detection of pigment and nutrient contents

At different growth stages, the pigment and nutrient contents of fruit leaves will change accordingly which will generate different reflection under light radiation. Spectral imaging records the spectral information of the target, which can be used to analyse growth conditions of the plant.

The Transformed Chlorophyll Absorption in Reflectance Index (TCARI) and Optimized Soil-adjusted Vegetation Index (OSAVI) usually be applied to minimize the effects of soil and LAI during pigment estimation. Zarco-Tejada et al. estimated the leaf carotenoid content of vineyards using UAV multispectral and hyperspectral images [78]. The combination of R515/R570 and TCARI/OSAVI indices could provide good prediction of carotenoid content. However, the results obtained with multispectral imagery yielded (R^2^ = 0.43) lower R^2^ values than those obtained with hyperspectral imagery (R^2^ = 0.48). A reason might be that multispectral cameras have independent lenses, resulting in errors in pixel matching in different wavebands.

Chlorophyll fluorescence is a probe for the study of photosynthesis, which can reflect the photochemical reaction process and is related to the chlorophyll content. The quantification of chlorophyll fluorescence aims to evaluate photosynthesis. The nonuniformity of the canopy will affect the measurement of the fluorescence signal. To extract the pure canopy fluorescence emission from the clustered pixels, the coverage range of each pixel should be fully considered [79]. Fraunhofer line depth (FLD) principle is the fundamental principle of chlorophyll fluorescence detection. Zarco-Tejada et al. captured multispectral images of a citrus orchard from a UAV [80]. Irradiance spectra at wavelengths of 763, 750 and 780 nm were selected as parameters of the model. They compared fluorescence retrieval models established by structural indices and chlorophyll index with FLD model and found that the prediction result of FLD model was obviously better.

N is the main mineral nutrient needed for chlorophyll production and other plant cell components (proteins, nucleic acids and amino acids) [81]. The determination of N can help with the timely management of nitrogen elements in the orchards to ensure growth vitality. Xuefeng et al. obtained spectral images of a citrus orchard at the height of 100 m above the canopy using a multispectral camera equipped on a UAV [82]. The camera had eleven spectral channels with wavelengths of 490, 550, 570, 671, 680, 700, 720, 800, 840, 900 and 950 nm. Mature and young leaf areas were selected manually in the images. The PLS model based on the original spectrum was the best prediction model for the total nitrogen content with R^2^ = 0.6469. The model that combined supported vector machine (SVM) and least square methods could estimate the starch content of mature leaves with R = 0.6822. In a red-blush pear orchard, Perry et al. used a six-band (at 550, 660, 710, 720, 730, 810 nm, and all bands were 10 nm wide) multispectral camera to collect images of the canopy with UAV [83]. They provided a new index, the Modified Canopy Chlorophyll Content Index (M3CI_710 nm), utilized for the assessment of canopy nitrogen. M3CI_710 nm was calculated according to the formula, (R_NIR_ + R_Red_ − R_RE_)/(R_NIR_ − R_Red_ + R_RE_), where R_NIR_ is the measured reflectance in the 810-nm band, R_Red_ is the measured reflectance in the 660-nm band, and R_RE_ is the measured reflectance in the 710-nm band. Regression results showed the highest R^2^ value (R^2^ = 0.67) for the leaf %N with the new index.

Spectral camera equipped on UAVs can capture canopy images of an orchard in a short time, but the flight is affected by air traffic control and battery power. Remote sensing satellites are man-made satellites used as remote sensing platforms in outer space, capable of covering the Earth or designated areas. Satellite data from remote sensing platforms can be used for agricultural research.

Multispectral sensors carried by satellites mainly include blue, green, red and NIR bands. Sentinel-2, which was launched by the European Space Agency, has sensor in the red-edge bands. Li et al. used Sentinel-2A remote sensing images to estimate the chlorophyll content of apple canopies [32]. The (NDVI_green_ + NDVI_red_ + NDVI_re_) was the best indices for the determination of chlorophyll content, and the SVM model provided better predictive results with R^2^ = 0.729 than back-propagation neural network (BPNN) method.

The above research results indicate that spectral imaging has great value in monitoring the pigment and nutrient contents of fruit trees. Satellite spectral remote sensing has a broad field of vision and can record macro features of large areas on the ground; nonetheless, the spatial resolution of the images is much lower than that of UAV images. The spectral imaging sensors carried by a UAV have more bands than satellite sensors. Thus, spectral imaging with a UAV is an available method for agricultural phenotypic research when time and space permit.

Compared with VIS–NIR spectroscopy, spectral imaging technology can obtain information more quickly and economize more labor force. It is noteworthy that spectral imaging cannot obtain spectral data directly, so complex image processing techniques are needed to extract spectral information from the images.

#### Detection of biochemical parameters of fruits

The experiments of fruit detection using spectral imaging are mainly carried out in the laboratory under controlled conditions including illumination, temperature, and distance [84–86]. Fruits were tested separately, which would take a long time when there are a large number of samples. Recently, on-the-go spectral imaging devices have been successfully applied in fruit detection [87].

Gutiérrez et al. installed a hyperspectral camera (400–1000 nm) on an all-terrain vehicle, to obtain dynamic hyperspectral images of a vineyard [87]. A relation matrix was established between all the pixels in the spectral image and the characteristic spectrum of the grape, the pixels with correlation coefficients that reached a predetermined value were selected as grape pixels. Epsilon-SVM algorithm was applied for the prediction of TSS (R^2^ = 0.91) and anthocyanin concentration (R^2^ = 0.72). The application of on-the-go hyperspectral imaging accomplished the detection of fruit components in the field, and the results could be compared with those under laboratory conditions.

Replacing all-terrain vehicles with field robotics, Wendel et al. implemented a driverless, automatic spectral scanner to predict the dry matter (DM) content of mangoes [71]. They developed an analytical method that unified the classification and regression analysis of hyperspectral images based on a convolutional neural network (CNN) and the PLS algorithm. The DM content prediction was not for individual fruit, but for the average over each tree. The prediction results revealed that the CNN model had a higher prediction accuracy (R^2^ = 0.64) than the PLS model (R^2^ = 0.58). To make a more accurate estimation of the mango yield, the research team counted the number of mangoes for each tree [88]. RGB images and hyperspectral images of mango trees were obtained simultaneously. After classifying the mango and non-mango pixels, the width and height of the local area of the mango pixels were parameterized to determine the local maximum. The number of mangoes was determined by the number of local maxima. The estimation of the mango counts showed that the accuracy of hyperspectral counting was lower than that of RGB imaging.

Although the resolution of RGB imaging is higher than that of spectral imaging, which is more conducive to image segmentation, spectral imaging can be applied in many aspects of phenotypic research, bringing much more information to researchers than RGB imaging. The estimation of the ripeness and the number of fruits by spectral imaging is beneficial for farmers to make a detailed harvest plan and maximize the benefits [88].

#### Detection of diseases

Plant diseases can cause considerable losses of plant quality and yield. Hence, effective identification methods should be adopted to prevent disease aggravation and infection [7]. Traditional detection methods are visual feature analysis and microbiological methods by laboratory experiments [89, 90]. However, these methods require specialized pathological knowledge and a long time to complete the detection process, resulting in the missing of the best opportunity for treatment. Non-invasive spectral imaging technology provides a rapid non-destructive testing method for plant disease detection. This section mainly focuses on the applications of hyperspectral and multispectral imaging for the disease detection of fruit trees in the field.

Verticillium wilt (VW) caused by the soil-borne fungus *Verticillium dahliae* Kleb is the most limiting disease in all traditional olive-growing regions worldwide. To detect VW, Calderón et al. captured airborne thermal, multispectral and hyperspectral images of a 7-ha commercial orchard. Through general linear model analysis, visible ratios (B/BG/BR) and fluorescence index (FLD3) were found to be effective in detecting VW at early stages of disease development [91]. To verify the applicability of spectral imaging methods in large-scale orchards, the research team carried out VW detection experiments in a 3000-ha commercial olive area. A manned aircraft replaced the UAV for image acquisition, since the UAV cannot be used in flight for a long time. Linear discriminant analysis (LDA) and SVM algorithms were used to classify healthy and diseased trees. For the whole data set, SVM expressed a high classification accuracy of 79.2%, while LDA achieved a classification accuracy of 59.0%. FLD3 was a good indicator that could identify olive trees at the early stages of disease development over as much at the orchard scale and even larger scales [92]. López-López et al. used the same analytical algorithms to detect red leaf blotch disease in an almond orchard [93]. Pigment indices (chlorophyll and carotenoid) and chlorophyll fluorescence can identify infected trees effectively in the early stage.

Laurel wilt (LW) is a lethal disease that spreads throughout the southeastern United States and has severely affected avocado industry. A digital colour camera was modified by adding a 37-mm filter ring to the front nose to capture images in the blue band (390–520 nm), green band (470–570 nm) and red-edge band (670–750 nm) [94]. The M-statistic was applied to evaluate the separability of healthy and diseased trees. According to the analysis of variance for the spectral images of the avocado canopy, B/G was found to be capable of separating the healthy trees from the laurel wilt-affected trees with M = 1.53. However, the researchers suggested using a high-spectral-resolution camera to improve the classification accuracy. A Tetracam mini-MCA-6 multispectral camera with six individual digital sensors (green: 580–10 nm; red: 650–10 nm, red-edge, Redge740: 740–10 nm, red-edge, Redge750: 750–10 nm, NIR760: 760–10 nm, and NIR850: 850–40 nm) was applied to obtain spectral images of an avocado orchard [95]. To make the tests more accurate, the researchers divided the degree of infection into four stages. The VIs TCARI_760–650_, NIR/G and redge/G, were able to discriminate LW at each developmental stage, and the value of M was up to 2.1. Although the modified digital camera had a significant reduction in cost, the multispectral camera had a higher number of bands and narrower bandwidth, so more spectral information could be applied for the classification of diseased trees to achieve an improved accuracy. Perez-Bueno et al. mounted a multispectral camera limiting the radiation to the bands at 560, 660 and 830 nm on a UAV [96]. ANN, logistic regression analysis (LRA), LDA and SVM were trained on NDVI to identify white root rot disease in avocado orchards. All four algorithms had the same resolution capability. The sensitivity of the LDA model was 55.5%, which is lower than that of the ANN and SVM models (78.6%). LRA had higher universality and a lower rate of false negatives than SVM in terms of classification. These conclusions can provide a reference for the selection of classification models.

When infected fruit trees show different response characteristics from healthy trees, spectral imaging technology can provide reliable information for the identification of infected fruit trees. Various forms of VIs can be indicators for identification. Effective identification of disease facilitates the implementation of healthy control and yield optimization measures, rather than relying on the chemical action of pesticides [90].

Multispectral cameras have separate sensors for each spectral band, and a multispectral image provides information on all pixels in the corresponding bands. Hyperspectral cameras adopt the push-sweep method to obtain all spectral information for all pixels in the bands [67]. The essence of a hyperspectral image is a cube composed of a large number of images, two dimensions are pixels, and the third dimension is the spectrum of each pixel [97]. For multispectral images, high precision is needed in pixel matching of images obtained from different sensors at the same time. We can conclude that spectral imaging is an effective method to realize contactless and spatially continuous monitoring for fruit tree phenotypic studies at the orchard level.

## Thermal imaging

Thermal imaging can produce digital images and draw a thermal map of the scene in false colour [98]. Traditionally, temperature is measured with thermometers, thermocouples, thermistors, and temperature detectors. These techniques are limited to the determination of specific points while thermal imaging enables continuous monitoring in space [99].

### The principle of thermal imaging

Everything in nature whose temperature is above absolute zero can emit infrared radiation, and this infrared radiation carries information about the characteristics of the object. Thermal motion of molecules or atoms will be more intense with increasing temperature, and the infrared radiation will also be enhanced [99]. The core of a thermal imaging camera is the infrared detector, which absorbs the infrared energy emitted by the object and converts it into voltage or current [100]. Thermal imaging technology can visualize the temperature information of the detected object, which has played an important role in the analysis of meteorological disaster management [101, 102], animal behaviour recognition [103, 104], and medical research [105, 106].

### The application of thermal imaging

The application of thermal imaging in the study of fruit tree phenotypes over recent years is summarized in Table 4. Some details and analysis are shown in the following section, especially focusing on the detection of water stress and disease.

**Table 4:** The applications of thermal imaging in the study of fruit tree phenotypes

Applications	Species	Scale	Spectral range	Devices	Detected parameters	Evaluation parameters		Advantages	Limitations	References
						Indices	Performance evaluation			
Water stress	Citrus orchard	T	7.5–13 µm	IR thermal camera	Ψ_s_	T_c _− T_a_	R^2^ = 0.42–0.76	Suitable for canopy temperature detection at the orchard level, reducing the deployment of a large number of temperature sensors	High cost; vulnerable to shadow, uneven lighting and other environmental effects	[108]
	Pear orchard	T	8–12 µm	FLIR 400	g_s_	T_c_				[107]
	Vineyard	O	7.5–13 µm	FLIR Tau II 320	P_n_	CWSI	R = − 0.80			[113]
		O	7.5–13 µm	FLIR Tau II 320	Ψ_s_	CWSI	R^2^ = 0.6931			[114]
					g_s_		R^2^ = 0.7061			
	Olive orchard	O	7.5–13.5 µm	Tau 2 324	Ψ_s_	CWSI	R^2^ = 0.60–0.73			[115]
					g_s_		R^2^ = 0.91			
		T	7.5–13 µm	ThermaCAM SC2000	Water status	CWSI				[110]
		T	8–14 µm	Flir One	Ψ_leaf_	T_c_	R^2^ = 0.81			[119]
						CWSI	R^2^ = 0.73			
	Almond orchard	O	8–12 µm	Miricle 307	Ψ_s_	CWSI	R^2^ = 0.67			[111]
						T_c _− T_a_	R^2^ = 0.65			
	Peach orchard	O	8–12 µm	Miricle 307	Ψ_s_	CWSI	R^2^ = 0.92			[111]
						T_c _− T_a_	R^2^ = 0.65			
	Apricot orchard	O	8–12 µm	Miricle 307	Ψ_s_	CWSI	R^2^ = 0.64			[111]
						T_c _− T_a_	R^2^ = 0.65			
Disease detection	Apple orchard	T	8–12 µm	Varioscan 3201 ST	Infected area of Scab disease	MTD	R^2^ = 0.85			[121]
					Severity levels		R^2^ = 0.71			
	Olive orchard	O	8–12 µm	Miricle 307	Severity levels of VW	T_c_ -T_a_	R^2^ = 0.76			[91]
						CWSI	R^2^ = 0.83			
		O	7.5–13 µm	FLIR SC655	Severity levels of VW	T_c _− T_a_				[92]
	Almond orchard	O	7.5–13 µm	FLIR SC655	Severity levels of Red leaf blotch	T_c _− T_a_				[93]

Note: In the “scale” column of the table, the fruit tree objects are divided into individual trees (T) and the whole orchard (O)

#### Detection of water stress

The lack of sufficient moisture in fruit trees can be considered water stress. Water stress is the most harmful environmental stress to the development and production of fruit trees and can affect cell division and vegetative growth. Water decreasing in plants leads to the photosynthetic rate subtracting and stomatal closure increasing, which result in a reduction of CO_2_ uptake and transpiration and thus a rise of plant temperature [107]. Although g_s_ cannot be directly measured by thermal imaging, it is feasible to measure canopy temperature (T_c_) to reflect stomatal status [108].

For the purpose of reducing the influence of field changes, Struthers et al. adjusted irrigation amount and conducted control experiments on 30 pear trees [107]. The stress treatment included 18 canopies and a control treatment of 12 canopies (normal irrigation). A long-wave thermal imager in 7.5–13 µm wavelength was attached to a mechanical lift. Thermal images of the canopy were acquired with a field of view of 25 degrees at nadir 1.3 m above the canopy. The results of multivariate analysis proved that T_c_ obtained by thermal imaging varied with g_s_, but this change may lag behind due to the influence of air temperature (T_a_) and vapor pressure deficit.

The Crop Water Stress Index (CWSI) is a reasonable quantitative evaluation parameter for crop water stress under evaporation pressure loss [109–111]. The CWSI can be calculated by the formula follows:

1 $$CWSI = \frac{{\left( {T_{c} - T_{a} } \right) - \left( {T_{c} - T_{a} } \right)_{ll} }}{{\left( {T_{c} - T_{a} } \right)_{ul} - \left( {T_{c} - T_{a} } \right)_{ll} }}$$

where T_c _− T_a_ represents the temperature difference between the crop canopy and the air; (T_c _− T_a_)_ul_ is the upper limit of (T_c _− T_a_), indicating that the canopy is immediately dried; and (T_c _− T_a_)_ll_ is the lower limit of (T_c _− T_a_), indicating the canopy under good irrigation conditions [112]. The estimations of (T_c _− T_a_)_ll_ and (T_c _− T_a_)_ul_ need to be careful and accurate, as they play important roles in the calculation.

Remote and proximal sensing measurements were compared with plant physiological variables by Matese et al. [113]. A small thermal imaging camera (7.5–13 µm) was mounted on a UAV as the remote sensing device, and images were collected at 70 m above the ground with a resolution of 9 cm/pixel. Proximal sensing images were collected at a 1.5 m distance from the lateral canopy with an infrared thermal imaging camera (8–14 µm). In the calculation of the CWSI, the researcher revised the formula as follows according to the actual situation:

2 $$CWSI = \frac{{T_{leaf} - T_{wet} }}{{T_{dry} - T_{wet} }}$$

T_dry_ and T_wet_ represent the temperature of a stressed leaf and an unstressed wet leaf, respectively, while T_leaf_ replaces T_c _− T_a_ indicating the leaf surface temperature. The leaves were treated with petroleum jelly or wetted to simulate the phenomenon of leaf stress and wetting. The results showed that remote sensing data had the same value as the proximal data. The CWSI value will increase when the net photosynthesis (P_n_) rate decreases under water stress. Therefore, the CWSI could be used as an indicator to evaluate the water status of the vineyard. In addition, the research team also detected the variation trend of the water state on a seasonal scale in the vineyard [114]. The CWSI correlated well with Ψ_s_ (R^2^ = 0.6931) and g_s_ (R^2^ = 0.7061). These results suggested that high-resolution thermal images can create great value for accurate vineyard management.

Egea et al. proposed a method to calculate the CWSI at different moments with Non-Water-Stressed Baselines (NWSBs) [115]. The NWSB was derived from T_c_ measured by infrared sensors mounted above olive trees, which is associated with weather changes such as solar radiation. The slope and intercept of the NWSB will change at different times in 1 day. To prevent the influence of rainy weather on leaf temperature and humidity, NWSB measurements were made only on continuous sunny days. This method is practical for simplifying the calculation of CWSI at different times. García-Tejero, IF et al. evaluated NWSBs in an orchard with three varieties of almonds [116]. It could be concluded from the results of the different varieties that the slopes of the NWSB were similar, but the intercepts were different. This conclusion also indicated that the NWSB intercept is related to weather conditions. The definition of the NWSB provides a reference for irrigation treatment under different water stress levels.

Thermal imagery is a spatial image with many mixed pixels, similar to spectral imagery, so separating the study area from the background is still the critical step in image processing. Moller et al. aligned a digital colour image with a thermal image and used the segmentation of the digital image as a mask, to perform a statistical analysis of the temperature in thermal images [117]. Agisoft PhotoScan was used to create a 3D point cloud and DEM using thermal images and GPS positions. Pixels of soil and leaves can be separated by determining the height threshold [113, 114]. All steps required a high level of image processing technology and related procedures. Salgadoe et al. proposed a method for automatically segmenting canopy pixels according to temperature histograms [118]. A histogram gradient threshold was set with a pre-defined local gradient to identify the highest and lowest canopy temperature. Compared with the segmentation methods for specific pixels, the thresholding segmentation method based on histograms is more time- and labour-saving and suitable for images of various resolutions, can be a reliable method for fast and standardized thermal analysis.

Although thermal cameras have contributed significantly to canopy temperature and water stress assessments, its cost is a burden for ordinary farmers. To reduce the cost of the camera, García-Tejero et al. used a thermal imaging camera connected to a smartphone (Flir One) and a conventional Thermal Imaging Camera Flir SC600 to capture images of almond trees [119]. The Flir One camera has a lower resolution (80 × 60 pixels) than the Flir SC600 (640 × 480 pixels). There was a strong similarity between T_c_ obtained by the Flir One camera and that measured by the Flir SC600 camera (R^2^ = 0.90), which indicated that the Flir One camera was available for water state assessment. The design of thermal imaging devices connected to mobile phones not only speeds up the monitoring process but also facilitates the use by fruit farmers.

Traditionally, plant water status is usually estimated by diffusion porometers or pressure chambers [6]. The manual measurement methods are not timely. Thermal imaging technology can analyse water status of fruit trees in a short time with the evaluation of T_c_. Using thermal imaging to monitor the spatial variation in orchard water status, the data from a large orchard area can be obtained quickly without installing an unreasonable number of on-site sensors. In addition, thermal imaging based on UAVs can be used to map the water status of the whole orchard, which can provide more detailed reference for the modulated irrigation strategy.

#### Detection of diseases

Plant disease pathogens may damage the cuticular cell structure of plant tissues, affect stomatal conductance and transpiration, and cause changes in leaf temperature [120]. The ability of thermal imaging to evaluate canopy temperature makes it possible to detect plant diseases.

Apple scab pathogen grows under the epidermis of apple leaves and absorbs nutrients from the subcuticular space and destroys the cuticle, causing water loss and temperature changes. Oerke et al. found significant differences in the thermal images corresponding to different stages of disease severity [121]. The maximum temperature difference (MTD) between the infected area and healthy area increased with the increase in the degree of infection. It was correlated with the infection area (R^2^ = 0.85) and overall infection severity (R^2^ = 0.71). *Polystigma amygdalinum* PF Cannon is also a fungus that lives on the surface of the leaf, causing almond trees to be infected with red leaf blotch disease. López-López et al. [93] collected thermal images in an almond orchard and found that the T_c _− T_a_ increased with the severity of the disease, especially in the stages of moderate or severe infection.

When a plant is affected by VW, the vascular system will be damaged, which impedes the flow of water, resulting in water stress [122, 123]. Calderón et al. identified the VW severity levels in olive orchards with airborne thermal imagery [91]. The g_s_ was measured in the leaf and near-canopy fields at the tree level, T_c_ and T_a_ were estimated from the thermal images. Measurement results showed that the T_c _− T_a_ would become higher and g_s_ would become lower as the severity level increased, which proved that crown temperature estimated with thermal imaging was effective in detecting VW in the early stage of disease development. The team then expanded the olive garden experiment by selecting nine areas in a larger commercial olive garden [92]. The nine areas covered different tree species, tree ages, planting densities and soil management techniques. The results showed that T_c _− T_a_ was still an effective indicator for VW detection in large-scale orchards.

The studies mentioned above suggested that the changes in T_c_ caused by disease can be monitored by thermal imaging techniques. Thermal imaging can help to separate healthy trees from infected trees, but it lacks diagnostic capability. It is difficult to determine whether the temperature change is caused by disease [121]. Combined with other imaging techniques to solve this problem is the focus of detecting fruit tree diseases at present.

## LiDAR scanning

Radar is an electronic device that transmits electromagnetic waves to the target and receives its echo, to obtain the distance and orientation from the target to the electromagnetic wave transmission point. LiDAR is a radar (radio detection and ranging) system that transmits a laser beam to detect the position, velocity and other characteristics of a target [124, 125].

### The principle of LiDAR

A LiDAR system consists of a single narrowband laser and a receiving system [126]. The laser fires a pulse of light at the target, and the reflected wave is picked up by the receiver. The receiver can accurately measure the propagation time of the light pulse from transmission to reflection. Light pulses travel at the speed of light, and the distance from the laser point to the target can be calculated based on the speed of light and the time of propagation. The position of the target can be determined according to the height and scanning angle of the laser [127].

### The application of LiDAR

Because of the ability to detect distance, LiDAR provides great value in estimating architecture parameters of fruit trees [128–131]. The application of LiDAR in phenotypic analysis has been reviewed by Colaço et al. [18]. This section mainly focuses on the combination of LiDAR and other technologies.

In the study of chlorophyll content, Ma et al. proposed a method to estimate the chlorophyll content in different areas of light intensity by using 3D models with colour characteristics [132]. A 3D laser scanner was used to acquire 3D data of apple trees; it was equipped with an internal colour camera that enabled the building of a colourful 3D model, and the colours represented different light intensities. They found that the colour index (R − B)/(R + B) was suitable for describing the chlorophyll content of different lights. Similarly, a fusion method of multispectral camera and 3D portable lidar images was proposed by Hosoi et al. [133]. The multispectral camera was placed on the points on the lines connecting the sample and LiDAR to promise the spectral images had the same angle of view as LiDAR data. The VI value of each pixel was added to lidar projection image as an additional attribute value reflecting spatial distribution of chlorophyll. This method provides both horizontal and vertical distribution of chlorophyll content over the canopy.

The uniting of LiDAR and colour imaging is beneficial to the detection of fruits. Underwood et al. used a mobile ground vehicle robot equipped with a 2D LiDAR and a machine vision camera to scan almond trees [134]. Within the LiDAR-based canopy mask, image classification was performed on the images associated with each tree for the estimation of canopy volume. To reduce the error caused by fruit occlusion, Stein et al. collected data from multiple viewpoints [135]. Based on spatial position coordinates, the fruits in the image were correlated with other viewpoint images to avoid double counting. The error between the number of fruits calculated by this method and the true value was 1.36%, which was considered to be a high precision.

In addition to precise 3D coordinates, LiDAR systems also record “intensity”, which is roughly defined as the backscattering intensity of the echo per test point and refers to the amplitude of the returned signal [125]. Different spectral reflectance properties will result in different backscattered intensification. Gené-Mola et al. converted the backscattering intensity at a laser wavelength of 905 nm into reflectance to separate the apple fruits from the canopy branches [136]. According to the feature that the reflectance value of apples is higher than that of leaves and branches at 905 nm wavelength, the correlation points corresponding to leaves and branches were removed from the point clouds, and remaining points were clustered to obtain the number of apples. The fusion result of reflectance information and LiDAR data was comparable to that of colour imagery. In terms of obtaining plant reflectance, LiDAR is less affected by illumination conditions than spectral imaging.

When the spatial information of orchards is detected with UAV or satellite imagery, the spatial resolution is limited by the flight altitude, and the observation angle is just overlooking. The integration of ground-based LiDAR with other technologies can facilitate the study of phenotypic characteristics from multiple lateral perspectives on fruit trees.

## Discussion

So far, much progress has been made in phenotypic study of fruit trees, but efforts still need to be made in the combination of technologies and the improvement of equipment. In further research, we should pay more attention to the practicability of technology so that we can make a real contribution to the development of agriculture. To this end, we proposed the following aspects for the focus and challenges of future fruit tree phenotypic research.

The applications of spectrometers and spectral imagers indicate that the changes in fruit tree pigment contents and water state can cause clear spectral responses in the VIS, NIR and short-wave infrared bands. However, hyperspectral sensors in the ultraviolet (UV) range have been demonstrated to detect salt stress in barley leaves [137]. UV–VIS spectroscopy has been used for the classification of tea types [138]. Whether the spectral information of the UV band or other bands is useful for the study of fruit tree phenotypes remains to be further verified in the future.

Cost reduction of optical imaging sensors will be the emphasis of the fruit tree phenotypic techniques, which can serve more farmers rather than scientists. The Flir One camera mentioned in part 5 is a good example [119], which has a lower cost than professional optical imaging devices and can satisfy the research demands in agriculture. Maintaining a high resolution while keeping a low cost is a challenge during the course of fabrication. In addition, it is necessary to develop image processing software with broad applied value so that mobile phones can replace computers to calculate the phenotypic characteristics of fruit trees.

LiDAR and imaging systems are complementary techniques for creating spatial coordinate descriptions and 3D image displays of plants [139]. LiDAR system provides precise elevation information, which is beneficial to the establishment of DSMs and DTMs. Wang et al. utilized airborne LiDAR and optical remote imagery to identify tree species in urban forests, and the classification accuracy was greatly improved compared with optical image analysis alone [74]. Consequently, in the study of fruit tree phenotypes, it may be a new method to identify fruit trees with airborne LiDAR and optical imaging.

## Conclusion

We attempted to review the non-destructive technologies applied in the field study of fruit tree phenotypes, including VIS–NIR spectroscopy, digital photography, multispectral and hyperspectral imaging, thermal imaging, and LiDAR. These techniques are feasible and valuable for the applications in phenotypic studies of fruit trees, such as the detection of architecture parameters, pigment and nutrient contents, water status, biochemical parameters of fruits, and plant disease. In particular, the combination of the data obtained by LiDAR and imaging techniques can promote the evaluation of phenotypic characteristics of fruit trees in three-dimensional space. Spatial characteristics have great contributions to the monitoring of spatial variability of pigment contents, the detection of fruit locations and the prediction of fruit yield.

The combination of non-destructive monitoring technology and automatic machinery realizes the automation of phenotypic research equipment. Ground-based devices are used for the detailed study of fruit trees at the tree level. However, it will take a long time to detect large orchard areas with terrestrial devices. Imaging techniques based on UAV and satellites have facilitated high-throughput phenotypic studies. The study of fruit tree phenotypes will be beneficial to rational irrigation, disease prevention, and yield improvement. Furthermore, phenotypic information can be considered the basis for screening excellent fruit tree species and promoting planting research on fruit trees.

## Data Availability

Not applicable.

## Abbreviations

ANN: Artificial neural network
BiPLS: Backward interval partial least squares
BPNN: Back-propagation neural network
CCD: Charge-coupled device
CMOS: Complementary metal oxide semiconductor
CNN: Convolutional neural network
Chl: Chlorophyll
Chl-b: Chlorophyll-b
Chl-a: Chlorophyll-a
Chl-t: Chlorophyll-t
C_v_: Crown volume
CWSI: Crop water stress index
DCP: Digital cover photography
DEM: Digital elevation model
DHP: Digital hemispherical photography
DM: Dry matter
DSM: Digital surface model
DTM: Digital terrain model
EWT: Equivalent water thickness
FD: First derivative
FLD: Fraunhofer line depth principle
FLD3: Fraunhofer line depth principle based on three spectral bands
FLDn: FLD3 normalized
GA: Genetic algorithm
GPS: Global positioning system
g_s_: Stomatal conductance
LA: Leaf area
LAI: Leaf area index
LDA: Linear discriminant analysis
LiDAR: Light detection and ranging
LRA: Logistic regression analysis
LW: Laurel wilt
M3CI: Modified canopy chlorophyll content index
MSI: Moisture spectral index
MTD: Maximum temperature difference
N: Nitrogen
NDGI: Normalized difference greenness vegetation index
NDVI: Normalized difference vegetation index
NDWI: Normalized difference water index
NIR: Near-infrared
NWSB: Non-Water-Stressed Baseline
OSAVI: Optimized Soil-adjusted Vegetation Index
PAI: Plant area index
PLS: Partial least squares
PLSR: Partial least squares regression
P_n_: Net photosynthesis
PRI: Photochemical reflectance index
UV: Ultraviolet
R: Red (spectral band of red)
R: Correlation coefficient (parameter of performance evaluation)
R^2^: Coefficients of determination
R_cv_: Cross validation correlation coefficient
RMSE: Root mean square error
RMSEP: Root mean square error prediction
RWC: Relative water content
SfM: Structure from Motion
SIPI: Structure Insensitive Pigment Index
Spec: Spectrometer
SVM: Supported vector machine
T_a_: Air temperature
TA: Titratable acidity
T_c_: Canopy temperature
TCARI: Transformed Chlorophyll Absorption in Reflectance Index
TSS: Total soluble solids
UAV: Unmanned aerial vehicle
VARI: Visible atmospherically resistant index
VI: Vegetation index
VIS: Visible
VIs: Vegetation indices
VW: Verticillium wilt
Ψ_leaf_: Leaf water potential
Ψ_pd_: Predawn leaf water potential
Ψ_s_: Stem water potential
